# The biochemical pattern defines MASLD phenotypes linked to distinct histology and prognosis

**DOI:** 10.1007/s00535-024-02098-8

**Published:** 2024-04-15

**Authors:** Javier Ampuero, Rocío Aller, Rocío Gallego-Durán, Javier Crespo, Jose Luis Calleja, Carmelo García-Monzón, Judith Gómez-Camarero, Joan Caballería, Oreste Lo Iacono, Luis Ibañez, Javier García-Samaniego, Agustín Albillos, Rubén Francés, Conrado Fernández-Rodríguez, Douglas Maya-Miles, Moisés Diago, Maria Poca, Raúl J. Andrade, Raquel Latorre, Francisco Jorquera, Rosa María Morillas, Desamparados Escudero, Manuel Hernández-Guerra, María Jesús Pareja-Megia, Jesús M. Banales, Patricia Aspichueta, Salvador Benlloch, José Miguel Rosales, Juan Turnes, Manuel Romero-Gómez, on behalf of HEPAmet Registry

**Affiliations:** 1 Hospital Universitario Virgen del Rocío, Universidad de Sevilla, Seville, Spain; 2 SeLiver Group, Instituto de Biomedicina de Sevilla, Seville, Spain; 3 Digestive Disease Department and CIBERehd, Virgen del Rocio University Hospital, Avenida Manuel Siurot S/N, 41013 Seville, Spain; 4 Centro de Investigación de Endocrinología y Nutrición, Hospital Clínico Universitario de Valladolid, Universidad de Valladolid, Valladolid, Spain; 5 Hospital Universitario Marqués de Valdecilla, Santander, Spain; 6 Hospital Universitario Puerta de Hierro, Madrid, Spain; 7 Liver Research Unit, Hospital Universitario Santa Cristina Instituto de Investigación Sanitaria Princesa Madrid, Madrid, Spain; 8 Hospital Universitario de Burgos, Burgos, Spain; 9 Liver Unit. Hospital Clínic. Institut d’Investigacions Biomèdiques August Pi I Sunyer (IDIBPAS), Barcelona, Spain; 10 Hospital Universitario Tajo, Aranjuez, Spain; 11 Instituto de Investigación Sanitaria Gregorio Marañón, Hospital General Universitario Gregorio Marañón, Madrid, Spain; 12 Hospital Universitario La Paz, IdiPAZ, Madrid, Spain; 13 Hospital Universitario Ramón y Cajal, Madrid, Spain; 14 Hospital General Universitario de Alicante, Universidad Miguel Hernández, Elche, Spain; 15 Hospital Universitario Fundación de Alcorcón, Universidad Rey Juan Carlos, Móstoles, Spain; 16 Hospital General Universitario de Valencia, Valencia, Spain; 17 Hospital de la Santa Creu i San Pau, Barcelona, Spain; 18 Unidad de Gestión Clínica de Enfermedades Digestivas, Instituto de Investigación Biomédica de Málaga-IBIMA-Plataforma BIONAND, Hospital Universitario Virgen de la Victoria, Universidad de Málaga, Malaga, Spain; 19 Hospital Universitari Son Llátzer, Mallorca, Spain; 20 Servicio de Aparato Digestivo, Complejo Asistencial Universitario de León, IBIOMED, León, España; 21 Hospital Germans Trias i Pujol, Badalona, Spain; 22 Hospital Clínico Universitario de Valencia, Universitat de València, Valencia, Spain; 23 Hospital Universitario de Canarias, Santa Cruz de Tenerife, Spain; 24 Pathology Department, Hospital Universitario Virgen de Valme, Sevilla, Spain; 25 Department of Liver and Gastrointestinal Diseases, Biodonostia Research Institute, Donostia University Hospital, University of the Basque Country (UPV/EHU), Ikerbasque, San Sebastian, Spain; 26 Department of Biochemistry and Genetics, School of Sciences, University of Navarra, Pamplona, Spain; 27 Biocruces Research Institute, Barakaldo, Department of Physiology, Faculty of Medicine and Nursing, University of Basque Country UPV/EHU, Leioa, Spain; 28 Servicio de Digestivo Hospital Arnau de Vilanova, Valencia, Spain; 29 Agencia Sanitaria Costa del Sol, Marbella, Spain; 30 Complejo Hospitalario Universitario de Pontevedra and IIS Galicia Sur, Pontevedra, Spain

**Keywords:** Hepatocellular, Cholestasis, MASLD, Phenotypes, Transaminases

## Abstract

**Background:**

MASLD can manifest as hepatocellular damage, which can result in mild elevation of aminotransferases. However, in some patients, MASLD presents with cholestatic pattern.

**Objective:**

To assess the impact of the biochemical pattern on the natural course of MASLD, including liver damage in histology, the accuracy of non-invasive tests(NITs), and prognosis.

**Methods:**

Multicenter study enrolling 2156 patients with biopsy-proven MASLD, who were classified based on their[ALT/ULN)]/[(ALP/ULN)] levels at the time of biopsy: (a) hepatocellular pattern(H), > 5; (b) mixed pattern(M),2–5; (c) cholestatic pattern(C), < 2. Outcomes: (a) histological evaluation of the single components of NAS, MASH, and fibrosis; (b) NITs and transient elastography assessing advanced fibrosis; (c) prognosis determined by the appearance of decompensated cirrhosis and death.

**Results:**

Out of the 2156 patients, 22.9% exhibited the H-pattern, whilst 31.7% exhibited the C-pattern. Severe steatosis, ballooning, lobular inflammation, and MASH (56.4% H *vs.* 41.9% M *vs.* 31.9% C) were more common in H-pattern (*p* = 0.0001),whilst C-pattern was linked to cirrhosis (5.8% H *vs.* 5.6% M *vs.* 10.9% C; *p* = 0.0001). FIB-4(0.74(95% CI 0.69–0.79) *vs.* 0.83 (95% CI 0.80–0.85); *p* = 0.005) and Hepamet Fibrosis Score(0.77 (95% CI 0.69–0.85) *vs.* 0.84 (95% CI 0.80–0.87); *p* = 0.044)exhibited lower AUROCs in the H-pattern. The C-pattern[HR 2.37 (95% CI 1.12–5.02); *p* = 0.024], along with age, diabetes, and cirrhosis were independently associated with mortality. Most patients maintained their initial biochemical pattern during the second evaluation.

**Conclusions:**

The H-pattern exhibited greater necro-inflammation in the histology than the C-pattern, whereas the latter showed more cirrhosis. The accuracy of NITs in detecting fibrosis was decreased in H-pattern. The occurrence of decompensated events and mortality was predominant in C-pattern. Therefore, identifying MASLD phenotypes based on the biochemical presentation could be relevant for clinical practice.

**Supplementary Information:**

The online version contains supplementary material available at 10.1007/s00535-024-02098-8.

## Introduction

Metabolic-associated-dysfunction steatotic liver disease (MASLD) is currently the most common cause of chronic liver disease worldwide, affecting 25% of the general population and more than 50% and 90% of diabetic and obese patients, respectively. In this scenario, MASLD is already the second leading cause of liver transplantation in the US, and it has increased from 1.2% in 2002 to 8.4% in 2016 in Europe [1]. In addition, the prevalence of hepatocellular carcinoma (HCC) is increasing exponentially in these patients, estimating that MASLD will be the leading cause of HCC shortly [2]. Taking it together, MASLD could be considered a pandemic, posing a significant health, social and economic burden [3].

Both non-invasive diagnosis [4] and stratification of patients with MASLD [5] represent a challenge, and multiple studies have tried to solve these questions. However, little attention has been paid to the biochemical presentation of this entity. The typical pattern of liver damage in patients with MASLD (hepatocellular lesion) is characterised biochemically by mild elevation of serum aminotransferases (AST and ALT) (known as hepatocellular pattern), usually no more than twice the upper limit of normal (ULN). By contrast, some MASLD patients show a cholestatic pattern based on elevated alkaline phosphatase (ALP) levels (associated with biliary damage), with or without elevated aminotransferases. Unfortunately, robust data extracted from large cohorts evaluating the impact of these patterns on the histology, diagnosis, and prognosis of MASLD is limited [6]. For instance, no studies have evaluated the implications of the MASLD-related biochemical pattern regarding the accuracy of non-invasive diagnosis to date.

A personalized approach based on MASLD phenotypes according to biochemical patterns has not been explored and could be of great interest since clinical trials persist ineffective in the context of MASLD [7]. Therefore, this study aimed to bring new insights into this complex disorder, enlightening the impact of MASLD phenotypes based on the biochemical pattern to enable a better stratification of the patients and a personalized strategy in the management of MASLD.

## Methods

### Selection of patients

This observational study enrolled 2156 patients with biopsy-confirmed MASLD, who were prospectively followed-up, from the Spanish HEPAmet Registry. This registry is governed by the Spanish Association for the Study of the Liver. Data monitoring is a fundamental element of the registry, ensuring data procurement accuracy and minimization of bias.

Patients underwent a liver biopsy according to the routine decisions in the clinical practice. The inclusion criterion was biopsy-proven MASLD, irrespective of the existence of MASH or fibrosis stage. Exclusion criteria were significant alcohol intake (> 30 g daily for men and > 20 g daily for women) and evidence of concomitant liver disease (i.e. viral hepatitis, autoimmune and cholestatic liver diseases, drug-induced fatty liver, hemochromatosis, or Wilson’s disease). The study was performed in agreement with the Declaration of Helsinki and approved by the Ethics and Clinical Research Committee of every center. All patients were informed of the nature of the study and gave their written consent to participate.

### Clinical assessment

Demographic characteristics, anthropometric measurements, and laboratory tests (ALT, AST, GGT, ALP, triglycerides, cholesterol, fasting glucose, insulin, creatinine, and albumin) were recorded at the same time as liver biopsy. An overnight (12 h) fasting blood sample was taken for routine biochemical analyses. Autoantibodies were obtained routinely to rule out autoimmune and cholestatic liver diseases. HOMA was calculated based on insulin and glucose (fasting insulin (mIU/mL) x fasting glucose (mg/mL) / 405). Furthermore, Hepamet Fibrosis Score (HFS) [1 / (1 + e [5.390–0.986 × Age [45–64 years of age]–1.719 × Age [≥ 65 years of age] + 0.875 × Male sex–0.896 × AST [35–69 IU/L]–2.126 × AST [≥ 70 IU/L]–0.027 × Albumin [4–4.49 g/dL]–0.897 × Albumin [< 4 g/dL]–0.899 × HOMA [2–3.99 with no DM]–1.497 × HOMA [≥ 4 with no DM]–2.184 × DM–0.882 × platelets × 1.000/µL [155–219]–2.233 × platelets × 1.000/µL [< 155])] [8], NAFLD Fibrosis Score (NFS) [9] and FIB-4 [(age × AST)/(Platelets × √ALT)] [10] were computed. Transient elastography (TE) was recorded at baseline, whose values were accepted if the success rate was > 60% and the interquartile range (IQR) was < 30% of the median value.

The biochemical pattern was defined according to the ratio ALT and ALP [ALT/ULN)] / [(ALP/ULN)] at the time of liver biopsy: (a) hepatocellular pattern (H), a ratio > 5; (b) mixed pattern (M), a ratio between 2 and 5; (c) cholestatic pattern (C), a ratio < 2. The ULN for ALT was 40 IU\L [11], whilst the ALP upper normal level was 130 IU/L. This approach was based on the ACG clinical guidelines [12].

Follow-up was defined as the time from the liver biopsy to the first event of cirrhosis complication (ascites, hepatic encephalopathy, variceal bleeding) or death. In the case of no event, patients were censored at 20 years of follow-up or at the end of the study period (May 2020).

### Histological assessment

The diagnosis of MASLD was based on histological criteria. All liver biopsies were assessed by experienced hepato-pathologists (led by MJPM) associated with the LITMUS histopathologists group [13], who were blinded regarding the patient’s evaluation and clinical data. Samples of < 15 mm in length or < 10 portal tracts were considered unsuitable for diagnosis and excluded. Several histological aspects were measured. Steatosis, lobular inflammation, and hepatocyte balloon degeneration were systematically assessed according to the NASH CRN Scoring System: (a) steatosis was rated as grade 0 (< 5%), grade 1 (5–33%), grade 2 (33–66%) and grade 3 (> 66%); (b) hepatocyte ballooning, was considered as 0 (none), 1 (mild-few) and 2 (moderate-marked); (c) lobular inflammation was rated as 0 (none), 1 (< 2 foci/20 optical field), 2 (2–4 foci/20 optical field), and 3 (> 4 foci/20 optical field). Although NASH CRN does not define MASH exactly using the NAS score [14], we determined MASH according to NAS score > 4 (with at least 1 point each in inflammation and ballooning) [15]. The severity of fibrosis was staged from 0 to 4.

### Objectives

We aimed to determine the impact of the biochemical pattern (hepatocellular *vs.* mixed *vs.* cholestatic) on the natural course and management of MASLD according to three objectives. First, analyzing the presence and the distribution of the single components of NAS (steatosis, inflammation, and ballooning), MASH, and fibrosis according to the biochemical pattern. Second, assessing the impact of the biochemical pattern on the accuracy of non-invasive tests (NITs) predicting advanced fibrosis. And third, performing an exploratory analysis to determine the prognosis of the three biochemical patterns in terms of the appearance of decompensated cirrhosis (first decompensated event) and death.

### Statistical analyses

Data were reported as the mean ± standard deviation for normal and median (interquartile range) for non-normal continuous variables, whilst frequency was used for discrete variables. In the univariate comparisons, we used the Student *t* test and ANOVA with Bonferroni adjustments for continuous samples and chi-square test or Fisher’s exact test for the qualitative ones. Non-parametric alternatives (Mann–Whitney *U* and Kruskal–Wallis tests) were used for non-normal distributions. The Kaplan–Meyer method assessed survival analysis and the log-rank test evaluated differences between patient subgroups.

Both logistic regression (cross-sectional analysis) and Cox proportional hazards model (longitudinal analysis) were used to estimate the odds ratio (OR) and hazard ratio (HR), respectively, and confidence intervals (CI). Variables that showed significance *p* < 0.05 in univariable analysis were entered into the backward regression analysis. They were constructed sequentially with variables introduced individually, and a significance level of *p* > 0.05 was used to remove the variables from the model.

On the other hand, the area under the ROC curve (AUROC) was computed to determine the diagnostic accuracy of the NITs for predicting advanced liver fibrosis, depending on the biochemical pattern.

The annual rate of the appearance of the first episode of decompensated cirrhosis and death was computed by dividing the number of patients with the defined event by the number of person-years of which patients were followed. We multiply rates by 100 to transform in cases per 100 person-years.

The method used for missing data was complete-case analysis since statistical packages excluded individuals with any missing value. STATA (12.0, STATA Corporation, College Station, TX, USA) statistical package was used in all analyses, and GraphPad Prism (version 6.0; GraphPad Software, Inc., La Jolla, CA) for graphics.

## Results

### Baseline features of the study population and according to the biochemical pattern

The baseline features of the study cohort are stated in Table 1. Briefly, the mean age was 51.4 ± 12.5 years old, with 47.6% (1026/2156) of males. The proportion of metabolic conditions was 35.2% (758/2156) type 2 diabetes mellitus (T2DM), 47% (1014/2156) arterial hypertension, 47.7% (1028/2156) hypercholesterolemia, 45.9% (990/2156) hypertriglyceridemia, and 61.8% (1333/2156) obesity. According to liver damage, steatohepatitis was present in 41% (884/2156) of the overall population, whilst the distribution of fibrosis was: F0 36.1% (779/2156), F1 26.5% (572/2156), F2 14.2% (306/2156), F3 13.3% (287/2156), and F4 7.1% (154/2156).

**Table 1:** Baseline features of the overall cohort, and according to the biochemical pattern

Characteristic	Overall cohort (*N* = 2156)	Hepatocellular pattern (*n* = 494)	Mixed pattern (*n* = 978)	Cholestatic pattern (*n* = 679)
Male sex	47.6% (1026/2156) #	45.5% (225/494)	50.5% (494/978)	44.9% (307/684)
Age; years ± SD	51.4 ± 12.5 *$	49.3 ± 12.7	51.8 ± 12.4	52.5 ± 12.4
BMI ± SD (kg/m2)	35.7 ± 9.1*#$	33.6 ± 7.7	35.5 ± 8.6	37.6 ± 10.1
Arterial hypertension	47.1% (1014/2152)	46.9% (230/490)	46.7% (457/978)	47.8% (327/684)
Type 2 diabetes mellitus	35.2% (758/2153)	36.6% (180/492)	35.6% (348/977)	33.6% (230/684)
Glucose ± SD (mg/dL)	117 ± 43#$	120 ± 46	118 ± 43	112 ± 42
Total cholesterol ± SD (mg/dL)	190 ± 45$	195 ± 49	190 ± 42	187 ± 45
Triglycerides ± SD (mg/dL)	160 ± 115$	169 ± 139	161 ± 105	150 ± 109
Hypertriglyceridemia	45.9% (990/2156) #$	50% (247/494)	48% (469/978)	40.1% (274/684)
AST ± SD (IU/L)	42 ± 39*#$	72 ± 63	38 ± 24	26 ± 15
ALT ± SD (IU/L)	59 ± 57*#$	115 ± 87	53 ± 27	27 ± 15
ALP ± SD (IU/L)	96 ± 59*#$	77 ± 48	89 ± 40	121 ± 77
GGT ± SD (IU/L)	104 ± 149$	117 ± 177	107 ± 131	91 ± 151
Bilirubin ± SD (mg/dL)	0.68 ± 0.4	0.72 ± 0.4	0.67 ± 0.4	0.67 ± 0.4
Albumin ± SD (g/dL)	4.36 ± 0.4#$	4.44 ± 0.4	4.39 ± 0.5	4.25 ± 0.5
Creatinine ± SD (mg/dL)	0.82 ± 0.3*	0.79 ± 0.2	0.82 ± 0.2	0.83 ± 0.3
Platelet count ± SD (× 10^9^/L)	233 ± 73	230 ± 63	231 ± 72	239 ± 81
Ferritin ± SD	203 ± 228*#$	279 ± 298	196 ± 194	156 ± 197
Steatohepatitis (%)	41.3% (884/2143) *#$	55.6% (273/491)	41% (399/973)	31.2% (212/679)
At-risk MASH (%)	20.4% (439/2143) *$	27.4% (135/491)	19.4% (190/977)	16.7% (114/679)
Cirrhosis (%)	7.3% (154/2098) #$	5.8% (28/482)	5.6% (54/956)	10.9% (72/660)
Hepatocellular carcinoma (%)	1.1% (19/1776)	0.7% (3/419)	1% (8/823)	1.5% (8/534)

*p value < 0.05 Hepatocellular vs. Mixed

^#^*p* value < 0.05 Mixed vs. Cholestatic

^$^*p* value < 0.05 Hepatocellular vs. Cholestatic

On the other hand, 22.9% (494/2156), 45.4% (978/2156), and 31.7% (684/2156) of patients showed a hepatocellular, mixed, and cholestatic pattern, respectively. Obviously, AST and ALT were predominant in the hepatocellular pattern (*p* = 0.0001), and ALP and GGT in the cholestatic pattern (*p* = 0.0001). We observed that patients with a hepatocellular pattern were significantly younger (*p* = 0.0001) and showed lower BMI (*p* = 0.0001) than individuals with mixed or cholestatic patterns. Despite the similar proportion of metabolic conditions, the cholestatic pattern was associated with lower levels of glucose (*p* = 0.010), triglycerides (*p* = 0.019), and total cholesterol (*p* = 0.012) compared with the hepatocellular pattern. In addition, albumin levels were increased in patients with a hepatocellular pattern (*p* = 0.0001), as well as an acute phase reactant like ferritin (*p* = 0.0001). The multivariate analyses showed that (excluding the variables defining the phenotypes, such as transaminases) age (OR 0.97 (95% CI 0.96–4.98); *p* = 0.0001), BMI (OR 0.96 (95% CI 0.94–4.98); *p* = 0.0001), albumin (OR 1.59 (95% CI 1.16–2.18); *p* = 0.004), and glucose (OR 1.004 (95% CI 1.001–1.007); *p* = 0.007) were independently associated with the hepatocellular pattern.

### Histological features depending on the biochemical pattern

**Fig. 1:**
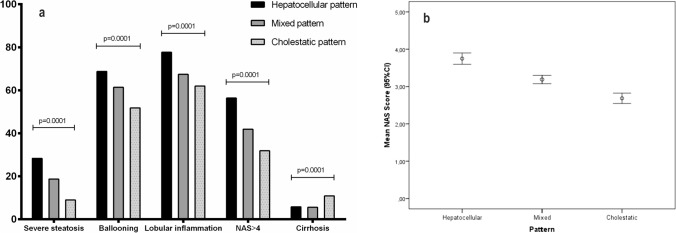
**A** Distribution of single components of NAS, steatohepatitis, and cirrhosis depending on the biochemical pattern. **B** NAS score according to the biochemical pattern

The distribution of single components of NAS and fibrosis was different considering the biochemical pattern. In this setting, severe steatosis (28.3% H *vs.* 18.7% M *vs.* 9% C; *p* = 0.0001), ballooning (68.8% H *vs.* 61.4% M *vs.* 51.8% C; *p* = 0.0001) and lobular inflammation (77.7% H *vs.* 67.4% M *vs.* 62% C; *p* = 0.0001) were more frequent in patients with a hepatocellular pattern (Fig. 1a). As a result, NAS was higher in patients with higher necro-inflammatory activity (3.7 ± 1.7 H *vs.* 3.2 ± 1.8 M *vs.* 2.7 ± 1.8 C; *p* = 0.0001) (Fig. 1b). Consequently, MASH (defined by NAS > 4) was also more frequently observed in patients with a hepatocellular pattern [(OR 2.23 (95%CI 1.63–3.05); *p* = 0.0001)] (Table 2). According to fibrosis, those patients with a cholestatic pattern showed a twofold risk of cirrhosis in comparison with the other patterns [(OR 2.77 (95%CI 1.54–4.99); *p* = 0.001) (Table 3)]. Also, the ratio [ALT/ULN)]/[(ALP/ULN)], considered as a continuous variable, was related to NAS > 4 and cirrhosis (Supplementary Tables 2 and 3, and Supplementary Fig. 1).

**Table 2:** A) Univariate and multivariate analyses to identify variables associated with steatohepatitis

Characteristic	NAS > 4 (*n* = 884)	NAS < 4 (*n* = 1259)	Univariate analysis (*p* value)	Multivariate analysis
Male sex	44.9% (397/884)	49.6% (624/1259)	0.034	
Age; years ± SD	52 ± 12.7	51.1 ± 12.4	0.078	OR 1.01 (95% CI 1.00–1.02); *p* = 0.020
BMI ± SD (kg/m2)	36.2 ± 8.7	35.3 ± 9.3	0.022	OR 1.03 (95% CI 1.01–1.04); *p* = 0.0001
Arterial hypertension	51.1% (451/882)	44.2% (555/1257)	0.001	
Type 2 diabetes mellitus	40.7% (359/882)	31.5% (396/1258)	0.0001	OR 1.24 (95% CI 1.00–1.54); *p* = 0.048
Glucose ± SD (mg/dL)	122 ± 45	113 ± 42	0.0001	
Total cholesterol ± SD (mg/dL)	193 ± 47	189 ± 43	0.098	
Triglycerides ± SD (mg/dL)	174 ± 119	149 ± 111	0.0001	OR 1.002 (95% CI 1.001–1.003); *p* = 0.0001
AST ± SD (IU/L)	49 ± 47	37 ± 31	0.0001	OR 1.01 (95% CI 1.003–1.012); *p* = 0.0001
ALT ± SD (IU/L)	68 ± 63	52 ± 51	0.0001	
ALP ± SD (IU/L)	95 ± 54	97 ± 62	0.281	
GGT ± SD (IU/L)	103 ± 140	106 ± 156	0.616	
Bilirubin ± SD (mg/dL)	0.67 ± 0.4	0.69 ± 0.4	0.450	
Albumin ± SD (g/dL)	4.37 ± 0.4	4.36 ± 0.4	0.417	
Creatinine ± SD (mg/dL)	0.81 ± 0.3	0.82 ± 0.2	0.317	
Platelet count ± SD (× 10^9^/L)	233 ± 73	234 ± 73	0.674	
Ferritin ± SD	217 ± 254	194 ± 206	0.057	
Biochemical pattern Hepatocellular Mixed Cholestatic	30.9% (273/884) 45.1% (399/884) 24% (212/884)	17.3% (218/1259) 45.6% (574/1259) 37.1% (467/1259)	0.0001	OR 2.23 (95% CI 1.63–3.05); *p* = 0.0001 OR 1.49 (95% CI 1.19–1.87); *p* = 0.001 Ref

**Table 3:** B) Univariate and multivariate analyses to identify variables associated with cirrhosis

Characteristic	Cirrhosis (*n* = 154)	No cirrhosis (*n* = 1944)	Univariate analysis (*p* value)	Multivariate analysis
Male sex	41.6% (64/154)	48.5% (942/1944)	0.099	OR 0.47 (95% CI 0.31–0.72); *p* = 0.0001
Age; years ± SD	59.6 ± 8.9	50.8 ± 12.6	0.0001	OR 1.03 (95% CI 1.01–1.05); *p* = 0.003
BMI ± SD (kg/m2)	34.4 ± 7.9	35.8 ± 9.2	0.055	
Arterial hypertension	62.7% (96/153)	46.1% (894/1944)	0.0001	
Type 2 diabetes mellitus	65.4% (100/154)	32.4% (630/1944)	0.0001	OR 3.17 (95% CI 2.07–4.85); *p* = 0.0001
Glucose ± SD (mg/dL)	143 ± 68	114 ± 40	0.0001	
Total cholesterol ± SD (mg/dL)	175 ± 45	192 ± 44	0.0001	
Triglycerides ± SD (mg/dL)	170 ± 216	159 ± 103	0.550	
AST ± SD (IU/L)	53 ± 40	41 ± 39	0.0001	
ALT ± SD (IU/L)	57 ± 73	59 ± 55	0.706	
ALP ± SD (IU/L)	126 ± 79	94 ± 57	0.0001	
GGT ± SD (IU/L)	177 ± 217	99 ± 138	0.0001	OR 1.002 (95% CI 1.001–1.003); *p* = 0.001
Bilirubin ± SD (mg/dL)	0.88 ± 0.7	0.67 ± 0.4	0.0001	OR 1.66 (95% CI 1.08–2.56); *p* = 0.020
Albumin ± SD (g/dL)	4.23 ± 0.6	4.37 ± 0.4	0.0001	
Creatinine ± SD (mg/dL)	0.83 ± 0.4	0.82 ± 0.2	0.834	
Platelet count ± SD (× 10^9^/L)	164 ± 72	239 ± 70	0.0001	OR 0.985 (95% CI 0.981–0.988); *p* = 0.0001
Ferritin ± SD	172 ± 219	207 ± 229	0.113	
Biochemical pattern Hepatocellular Mixed Cholestatic	18.2% (28/154) 35.1% (54/154) 46.8% (72/154)	23.4% (454/1944) 46.4% (902/1944) 30.2% (588/1944)	0.0001	Ref OR 1.10 (95% CI 0.61–1.99); *p* = 0.755 OR 2.77 (95% CI 1.54–4.99); *p* = 0.001

### Accuracy of non-invasive tests according to the biochemical pattern

**Fig. 2:**
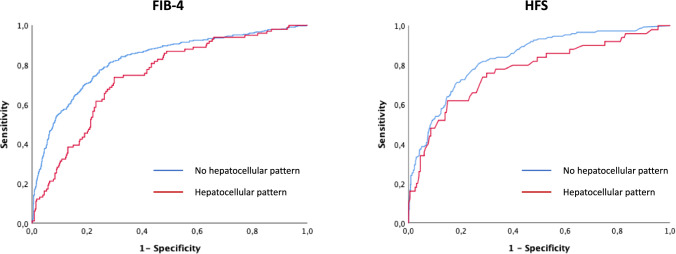
Accuracy of FIB-4 and HFS predicting advanced fibrosis according to the biochemical pattern

**Fig. 3:**
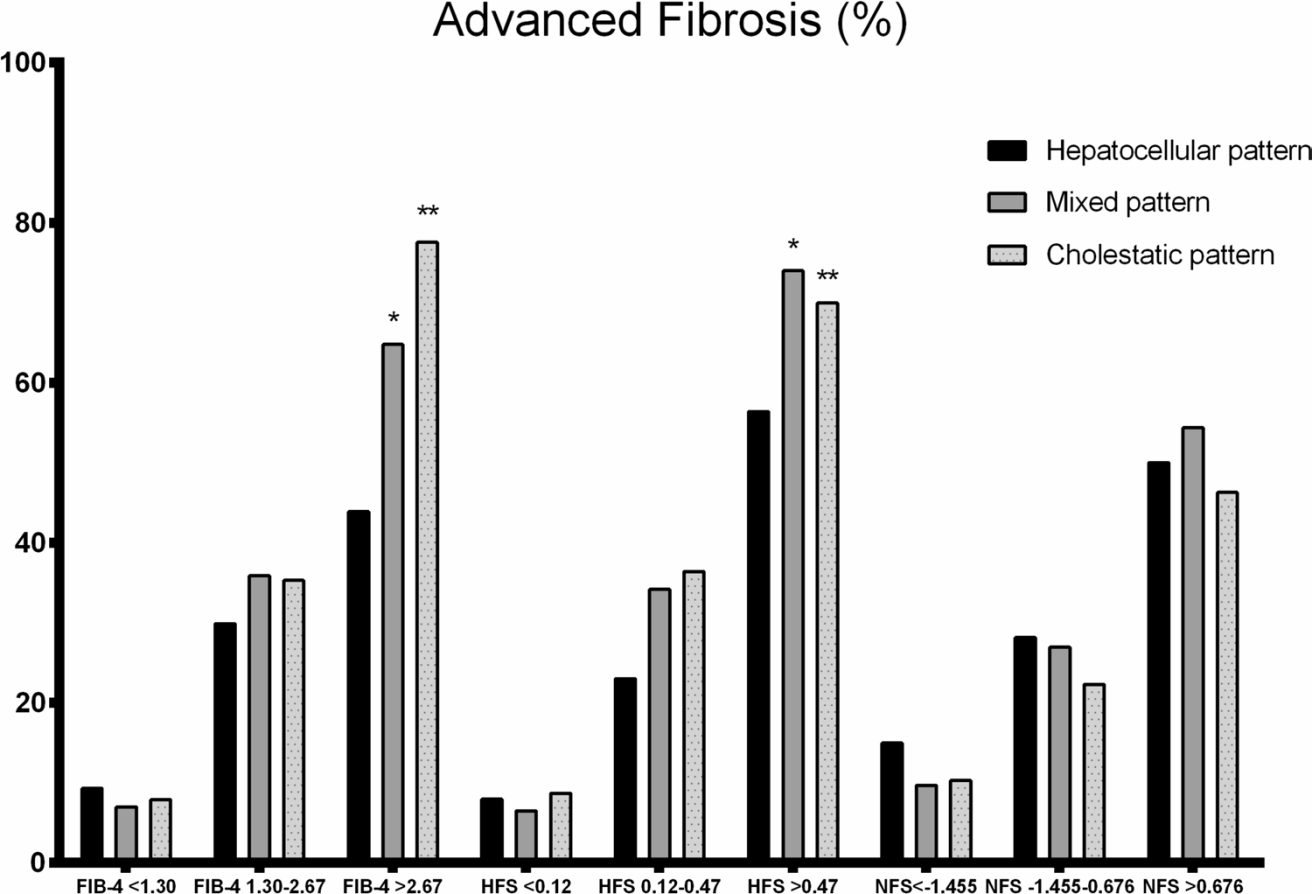
Prediction of advanced fibrosis according to the thresholds of NITs

We assessed the accuracy of different NITs and transient elastography predicting advanced fibrosis according to the biochemical pattern. Both FIB-4 (0.74 H (95% CI 0.69–0.79) *vs.* 0.83 (95% CI 0.80–0.85) no H; *p* = 0.005) and HFS (0.77 H (95% CI 0.69–0.85) *vs.* 0.84 (95% CI 0.80–0.87) no H; *p* = 0.044) showed lower AUROCs in the presence of a hepatocellular pattern (Fig. 2), whilst this fact did not occur with NFS (0.73 H (95% CI 0.67–0.79) *vs.* 0.77 (95% CI 0.74–0.80) no H; *p* = 0.217). Particularly, the prediction of advanced fibrosis was affected in patients showing values above the higher threshold of NITs when the hepatocellular pattern was present (Fig. 3). However, transient elastography had a similar performance irrespective of the pattern (0.78 H (95% CI 0.72–0.85) *vs.* 0.79 (95% CI 0.75–0.83) no H; *p* = 0.840).

### Prognostication based on the biochemical pattern

**Fig. 4:**
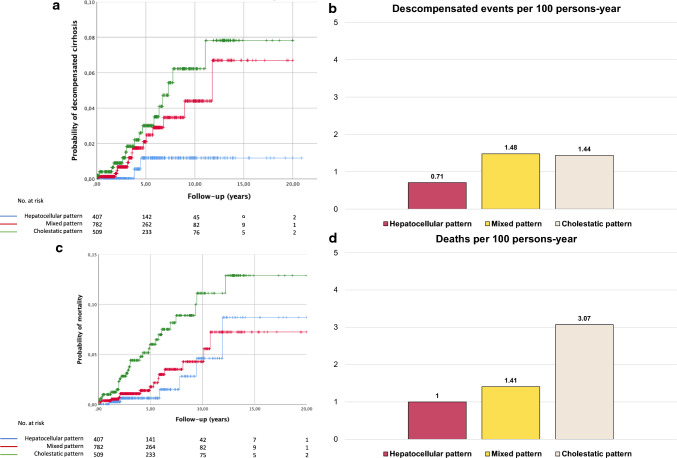
**A** Cumulative probability for decompensated cirrhosis. **B** Annual incidence rate of decompensated cirrhosis. **C** Cumulative probability for mortality. **D** Annual incidence rate of mortality

In the longitudinal study, 1776 subjects were considered because some patients were included in clinical trials or underwent bariatric surgery. During the follow-up (mean 4.6 years, IQR 1.5–6.6), 1.9% (33/1776) of patients suffered from some decompensation event (0.8% hepatic encephalopathy, 1.5% ascites, 0.7% variceal bleeding), 1.1% (19/1776) had hepatocellular carcinoma, and 3.2% (56/1776) died. First, patients showing a hepatocellular pattern suffered from less decompensated cirrhosis than the other groups (3.1% (16/509) C *vs.* 1.8% (14/782) M *vs.* 0.7% (3/407) H; LogRank 6.104; *p* = 0.047) (Fig. 4a). Particularly, cirrhotic patients with a hepatocellular pattern showed a lower annual decompensation rate than those with other patterns (Fig. 4b). Second, the cholestatic pattern showed higher mortality during the follow-up (5.7% (29/509) C *vs.* 2.3% (18/782) M *vs.* 2.2% (9/407) H; LogRank 8.828; *p* = 0.012) (Fig. 4c). In the case of cirrhotic patients, the annual incidence of mortality was significantly higher in subjects with a cholestatic pattern (Fig. 4d). The cause of mortality was similar between groups considering hepatic and extrahepatic fatal events. In the multivariate analysis, a cholestatic pattern [HR 2.37 (95% CI 1.12–5.02); *p* = 0.024], age [HR 1.07 (95% CI 1.05–1.10); *p* = 0.0001], T2DM [HR 2.04 (95% CI 1.08–3.82); *p* = 0.027], and cirrhosis [HR 4.36 (95% CI 2.36–8.01); *p* = 0.0001] were independently associated with mortality (Table 4).

**Table 4:** Univariate and multivariate analyses for predicting mortality in the longitudinal cohort

Characteristic	Mortality (*n* = 56)	Survival (*n* = 1720)	Univariate analysis (*p* value)	Multivariate analysis
Male sex	41.1% (23/56)	49.9% (858/1720)	0.194	
Age; years ± SD	59.9 ± 10.4	52 ± 12.6	0.0001	HR 1.07 (95% CI 1.05–1.10); *p* = 0.0001
BMI ± SD (kg/m2)	33.6 ± 7.4	34.7 ± 8.8	0.426	
Arterial hypertension	53.6% (30/56)	46.6% (799/1716)	0.301	
Type 2 diabetes mellitus	60.7% (34/56)	35.8% (614/1717)	0.0001	HR 2.04 (95% CI 1.08–3.82); *p* = 0.027
Glucose ± SD (mg/dL)	144 ± 67	118 ± 43	0.006	
Total cholesterol ± SD (mg/dL)	187 ± 61	191 ± 43	0.676	
Triglycerides ± SD (mg/dL)	189 ± 200	161 ± 117	0.314	
Hypertriglyceridemia	37.5% (21/56)	47.3% (813/1720)	0.150	
AST ± SD (IU/L)	53 ± 56	43 ± 40	0.076	
ALT ± SD (IU/L)	64 ± 113	62 ± 57	0.819	
ALP ± SD (IU/L)	133 ± 110	97 ± 58	0.018	
GGT ± SD (IU/L)	159 ± 213	110 ± 151	0.095	
Bilirubin ± SD (mg/dL)	0.80 ± 0.5	0.69 ± 0.4	0.104	
Albumin ± SD (g/dL)	4.20 ± 0.7	4.39 ± 0.4	0.061	
Creatinine ± SD (mg/dL)	0.88 ± 0.4	0.82 ± 0.2	0.030	
Platelet count ± SD (× 10^9^/L)	207 ± 88	233 ± 73	0.036	
Ferritin ± SD	212 ± 308	216 ± 239	0.908	
Steatohepatitis	41.1% (23/56)	40.6% (699/1720)	0.948	
Cirrhosis	25.9% (14/54)	7.8% (132/1698)	0.0001	HR 4.36 (95% CI 2.36–8.01); *p* = 0.0001
Biochemical pattern Hepatocellular Mixed Cholestatic	16.1% (9/56) 32.1% (18/56) 51.8% (29/56)	23.8% (410/1720) 46.8% (805/1720) 29.4% (505/1720)	0.002	Ref HR 1.14 (95% CI 0.51–2.55); *p* = 0.747 HR 2.37 (95% CI 1.12–5.02); *p* = 0.024

### Dynamic changes over time in the biochemical pattern

Up to 845 patients had at least a second evaluation of the biochemical pattern during the follow-up. More than half of the patients maintained the initial biochemical pattern, whilst only a few changed from hepatocellular to cholestatic pattern or vice versa (Supplementary Table 1). In these patients, a trend for lower mortality in subjects with both initial and final hepatocellular pattern (0.9% (1/117)) compared with those with final mixed (2.8% (2/72)) and cholestatic patterns (6.7% (1/15)) was observed. On the other hand, patients who maintained the cholestatic pattern during the follow-up showed higher mortality (5.9% (7/118) than those who finally changed for any of the other patterns (1% (1/98) (*p* = 0.07).

## Discussion

Individuals exhibiting mild-to-moderate cholestasis in the context of MASLD presented diverse signs and clinical features compared to those with a hepatocellular pattern, showing additional important issues regarding diagnosis and prognosis. In this setting, some relevant aspects must be emphasized. First, the cholestatic pattern was shown as often as the hepatocellular pattern in the overall MASLD cohort. Second, individuals showing a hepatocellular pattern had more necroinflammatory activity in the histology compared with those with a cholestatic pattern, whilst this latter showed more frequently cirrhosis. Third, the accuracy of NITs detecting advanced fibrosis, particularly FIB-4, and HFS, was decreased in the presence of a hepatocellular pattern. And fourth, the prognosis was worse in patients with a predominant cholestatic pattern. Therefore, we should consider different MASLD phenotypes according to the biochemical presentation, which is easy to identify, with significant consequences for the making-decision process in clinical practice.

MASLD is an entity typically associated with a hepatocellular pattern; this is a predominant elevation of transaminases (AST and ALT) instead of enzymes of cholestasis (particularly ALP). In this setting, little is known about the prevalence of the cholestatic pattern in MASLD. About an one-fourth of patients in the HEPAmet registry (one of the largest registries worldwide) showed a cholestatic pattern, whilst other studies have reported between 27 and 43% [6, 16]. Besides, we observed some baseline features that differ between the patterns beyond the liver profile. Interestingly, the cholestatic pattern was associated with older age and higher BMI, and individuals showing a hepatocellular pattern had higher levels of triglycerides, cholesterol, and glucose, despite the rates of metabolic conditions (i.e. T2DM or arterial hypertension) were similar. While the association between age and BMI with the cholestatic pattern has been reported previously, the relationship between a worse control of metabolic factors and the hepatocellular pattern is novel [6, 16, 17]. This latter could be explained, at least in part, by the fact that some metabolic conditions (such as T2DM) can promote liver inflammation [18].

The cholestatic pattern has been associated with a higher liver fibrosis stage by an Italian study in 582 patients (only 435 with biopsy-proven MASLD) [6] and an Israeli study in 106 patients [16]. However, they evaluated neither ballooning nor lobular inflammation in the liver biopsies. Our study represents the largest cohort (*n* = 2156) of biopsy-proven MASLD patients evaluating the histology depending on the biochemical pattern. In this setting, we observed that the single components of NAS (steatosis, lobular inflammation, and ballooning) were significantly more frequent in patients with a hepatocellular pattern, and, consequently, they had higher NAS and more presence of MASH. These findings are not surprising, given that this pattern is defined by a predominant elevation of ALT and AST, which usually has been associated with a higher necro-inflammation in the histology. By contrast, patients with a cholestatic pattern doubled the risk of cirrhosis compared to the mixed and hepatocellular pattern, and, probably, this fact explains the lower percentage of MASH [19] and albumin levels in the presence of cholestasis. Cholestasis secondary to chronic liver injury may denote a more severe disease course and development of end-stage liver disease [20]. In a small subgroup of subjects stratified for these patterns, a significant down-expression of NR1H3, RXRα and VCAM 1 genes was found in patients with cholestatic compared to those with the hepatocellular pattern [6]. Thus, a MASLD patient with a cholestatic pattern, despite showing normal transaminases, should be monitored to look for advanced fibrosis more carefully than others.

The role of the biochemical pattern on the prognosis of MASLD patients has not yet been assessed. An Italian study observed a higher risk of decompensated events in patients with a cholestatic pattern (considering that they included cirrhotic patients diagnosed by clinical features) [6]. At the same time, no studies have assessed the impact on mortality or the dynamic changes of the biochemical patterns over time. In our study, more than 1,700 patients were followed-up to evaluate these questions. First, we observed that patients with a hepatocellular pattern showed lower rate of decompensated cirrhosis, in both the overall and the cirrhotic cohorts, than in the absence of it. Second, we observed that the cholestatic pattern was independently related to higher mortality, together with older age, and the presence of T2DM and cirrhosis. Interestingly, the annual incidence of mortality was three times higher in patients with predominant ALP levels compared to those with a hepatocellular pattern. Third, we observed that patients with initial and/or final hepatocellular pattern showed about 1% of mortality during the follow-up, whilst patients with initial and/or final cholestatic pattern died around 6%. Of note, the leading cause of mortality was similar between the groups despite the hepatocellular pattern had more prevalence of MASH (typically associated with higher cardiovascular-related mortality [21]) and patients with a cholestatic pattern had more prevalence of cirrhosis (usually related to liver-related mortality [22]).

The use of NITs, including FIB-4, HFS, and NFS, is recommended in clinical practice by international guidelines [23–25]. Despite they have robust thresholds to detect advanced fibrosis [26], there are some circumstances, such as age, in which they must be modified to increase the specificity or the sensitivity [27]. The impact of the biochemical pattern on the accuracy of NITs has not been assessed yet in MASLD patients. Overall, FIB-4 and HFS were superior to NFS in detecting advanced fibrosis. However, we observed a significantly decreased accuracy for FIB-4 and HFS in patients showing a hepatocellular pattern. Of note, the identification of advanced fibrosis in patients above the higher threshold was particularly affected and significantly decreased compared to those without a hepatocellular pattern. These results should make us to reconsider the higher cut-offs for FIB-4 and HFS to avoid a relevant number of patients with false positive results. On the other hand, we also included patients who underwent transient elastography, although the biochemical pattern did not affect this technique.

We must recognize that the current study shows some limitations. First, despite collecting all the patients’ medications for the different comorbidities, we cannot exclude completely that some could show a drug-induced cholestatic pattern. However, the fact of including more than two thousand patients and selecting a high specificity ALP/ALT ratio decreases this likelihood dramatically. Second, the dynamic changes in the biochemical pattern were assessed in half of the longitudinal cohort. However, we consider valuable this exploratory analysis (very few patients transiting from a hepatocellular to a cholestatic pattern or vice versa) because there are no published data about the durability of the pattern in MASLD. And third, this study was not designed to perform any experimental approach to explain the underlying mechanisms of cholestasis in MASLD. However, two studies have previously identified specific features in patients with this pattern, including morphological changes and a different gene expression [6, 17]. Some drugs are being tested simultaneously for MASLD and primary biliary cholangitis (i.e. obeticholic acid, elafibranor) with a suboptimal efficacy in the presence of fatty liver, so working on the biological plausibility of some grade of cholestasis in MASLD is very attractive.

In conclusion, the current study lights different MASLD phenotypes that the biochemical pattern could easily identify. Selecting patients to undergo biopsy and anticipate liver damage, carefully monitoring at-risk patients of cirrhosis and decompensation, or receiving personalized experimental therapy in the setting of clinical trials are some of the potential clinical consequences of our research. Further studies are warranted to confirm these results and, mainly, to analyze the underlying mechanisms that explain the clinical differences between the MASLD phenotypes and guide to a specific therapeutic approach.

### Supplementary Information

Below is the link to the electronic supplementary material.

Supplementary file1 (TIF 8751 KB)

Supplementary file2 (DOCX 18 KB)

Supplementary file3 (DOCX 18 KB)

Supplementary file4 (DOCX 13 KB)

## Abbreviations

AHT: Arterial hypertension.
ALP: Alkaline phosphatase.
AUROC: Area under the ROC curve.
BMI: Body mass index.
CI: Confidence interval.
HCC: Hepatocellular carcinoma.
HR: Hazard ratio.
MASLD: Metabolic-associated-dysfunction steatotic liver disease.
MASH: Metabolic-associated steatohepatitis.
NIT: Non-invasive tests.
OR: Odds ratio.
T2DM: Type 2 diabetes mellitus.
ULN: Upper limit of normal
