# Supplementary figures and images for: The biochemical pattern defines MASLD phenotypes linked to distinct histology and prognosis
Source: J Gastroenterol. 2024 Apr 15;59(7):586–97. doi: 10.1007/s00535-024-02098-8 (PMC11217049; doi:10.1007/s00535-024-02098-8)

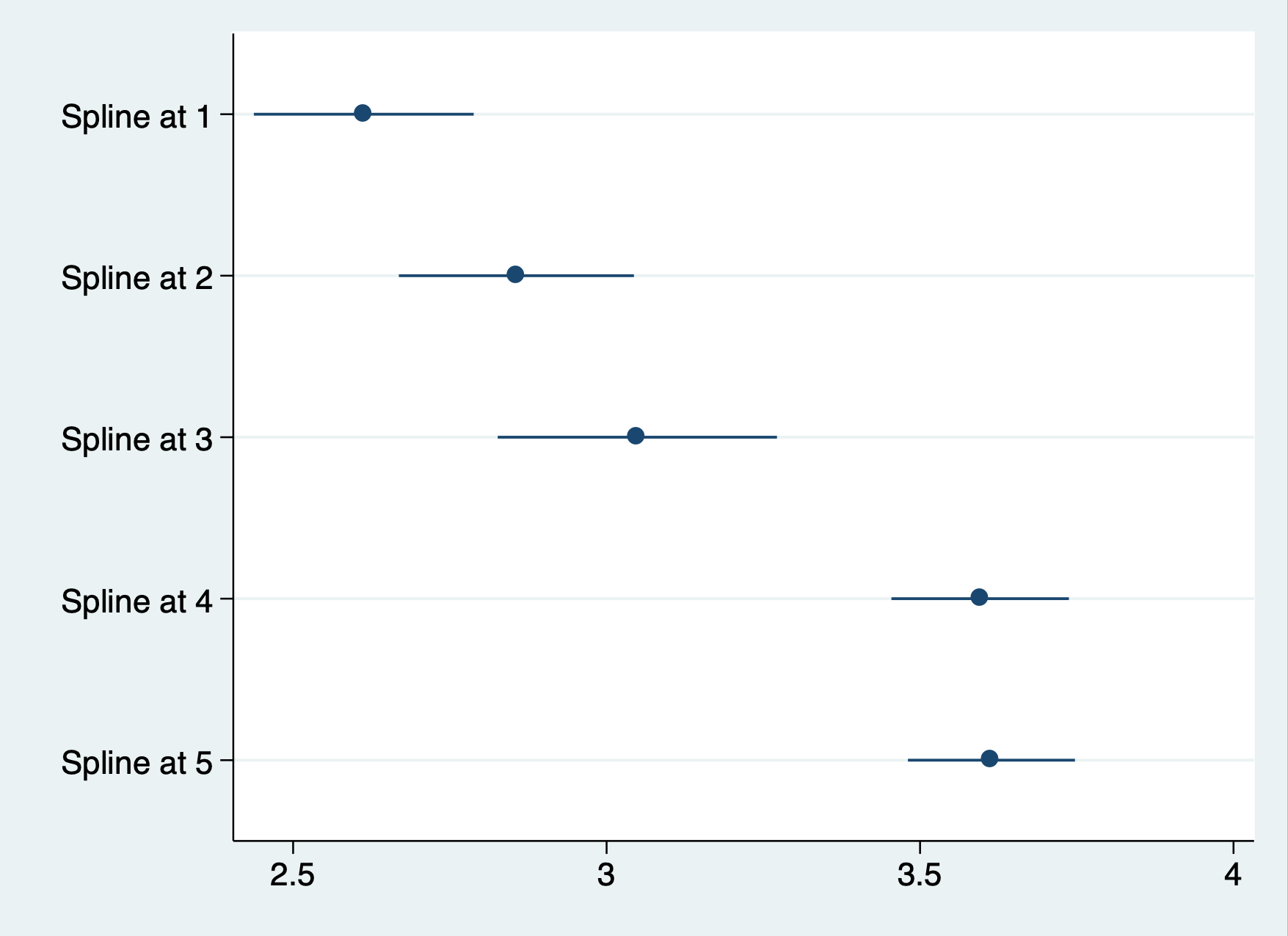

Supplement: Supplementary file 1 — Supplementary file1 (TIF 8751 KB) [file 535_2024_2098_MOESM1_ESM.tif]
