# Supplementary material for: The biochemical pattern defines MASLD phenotypes linked to distinct histology and prognosis
Source: J Gastroenterol. 2024 Apr 15;59(7):586–97. doi: 10.1007/s00535-024-02098-8 (PMC11217049; doi:10.1007/s00535-024-02098-8)
Supplement: Supplementary file 2 — Supplementary file2 (DOCX 18 KB) [file 535_2024_2098_MOESM2_ESM.docx]

| **Characteristic** | **NAS>4 (n=884)** | **NAS<4 (n=1259)** | **Univariate analysis**  **(p value)** | **Multivariate analysis** |
| --- | --- | --- | --- | --- |
| Male sex | 44.9% (397/884) | 49.6% (624/1259) | 0.034 |  |
| Age; years ± SD | 52 ± 12.7 | 51.1 ± 12.4 | 0.078 | OR 1.01 (95%CI 1.002-1.02); p=0.011 |
| BMI ± SD (kg/m2) | 36.2 ± 8.7 | 35.3 ± 9.3 | 0.022 | OR 1.02 (95%CI 1.01-1.04); p=0.0001 |
| Arterial Hypertension | 51.1% (451/882) | 44.2% (555/1257) | 0.001 |  |
| Type 2 Diabetes Mellitus | 40.7% (359/882) | 31.5% (396/1258) | 0.0001 | OR 1.25 (95%CI 1.01-1.55); p=0.039 |
| Glucose ± SD (mg/dL) | 122 ± 45 | 113 ± 42 | 0.0001 |  |
| Total cholesterol ± SD (mg/dL) | 193 ± 47 | 189 ± 43 | 0.098 |  |
| Triglycerides ± SD (mg/dL) | 174 ± 119 | 149 ± 111 | 0.0001 | OR 1.002 (95%CI 1.001-1.003); p=0.0001 |
| AST ± SD (IU/L) | 49 ± 47 | 37 ± 31 | 0.0001 | OR 1.006 (95%CI 1.001-1.01); p=0.014 |
| ALT ± SD (IU/L) | 68 ± 63 | 52 ± 51 | 0.0001 |  |
| ALP ± SD (IU/L) | 95 ± 54 | 97 ± 62 | 0.281 |  |
| GGT ± SD (IU/L) | 103 ± 140 | 106 ± 156 | 0.616 |  |
| Bilirubin ± SD (mg/dL) | 0.67 ± 0.4 | 0.69 ± 0.4 | 0.450 |  |
| Albumin ± SD (g/dL) | 4.37 ± 0.4 | 4.36 ± 0.4 | 0.417 |  |
| Creatinine ± SD (mg/dL) | 0.81 ± 0.3 | 0.82 ± 0.2 | 0.317 |  |
| Platelet count ± SD (x 10^9^/L) | 233 ± 73 | 234 ± 73 | 0.674 |  |
| Ferritin ± SD | 217 ± 254 | 194 ± 206 | 0.057 |  |
| [ALT/ULN / ALP/ULN] ratio | 4.46 ± 5.3 | 3.38 ± 3.15 | 0.0001 | OR 1.11 (95%CI 1.07-1.16); p=0.0001 |
