# Supplementary material for: The biochemical pattern defines MASLD phenotypes linked to distinct histology and prognosis
Source: J Gastroenterol. 2024 Apr 15;59(7):586–97. doi: 10.1007/s00535-024-02098-8 (PMC11217049; doi:10.1007/s00535-024-02098-8)
Supplement: Supplementary file 3 — Supplementary file3 (DOCX 18 KB) [file 535_2024_2098_MOESM3_ESM.docx]

| **Characteristic** | **Cirrhosis (n=154)** | **No cirrhosis (n=1944)** | **Univariate analysis**  **(p value)** | **Multivariate analysis** |
| --- | --- | --- | --- | --- |
| Male sex | 41.6% (64/154) | 48.5% (942/1944) | 0.099 | OR 0.48 (95%CI 0.31-0.73); p=0.001 |
| Age; years ± SD | 59.6 ± 8.9 | 50.8 ± 12.6 | 0.0001 | OR 1.03 (95%CI 1.01-1.05); p=0.003 |
| BMI ± SD (kg/m2) | 34.4 ± 7.9 | 35.8 ± 9.2 | 0.055 |  |
| Arterial Hypertension | 62.7% (96/153) | 46.1% (894/1944) | 0.0001 |  |
| Type 2 Diabetes Mellitus | 65.4% (100/154) | 32.4% (630/1944) | 0.0001 | OR 2.95 (95%CI 1.94-4.49); p=0.0001 |
| Glucose ± SD (mg/dL) | 143 ± 68 | 114 ± 40 | 0.0001 |  |
| Total cholesterol ± SD (mg/dL) | 175 ± 45 | 192 ± 44 | 0.0001 |  |
| Triglycerides ± SD (mg/dL) | 170 ± 216 | 159 ± 103 | 0.550 |  |
| AST ± SD (IU/L) | 53 ± 40 | 41 ± 39 | 0.0001 |  |
| ALT ± SD (IU/L) | 57 ± 73 | 59 ± 55 | 0.706 |  |
| ALP ± SD (IU/L) | 126 ± 79 | 94 ± 57 | 0.0001 |  |
| GGT ± SD (IU/L) | 177 ± 217 | 99 ± 138 | 0.0001 | OR 1.002 (95%CI 1.001-1.003); p=0.001 |
| Bilirubin ± SD (mg/dL) | 0.88 ± 0.7 | 0.67 ± 0.4 | 0.0001 | OR 1.64 (95%CI 1.08-2.49); p=0.021 |
| Albumin ± SD (g/dL) | 4.23 ± 0.6 | 4.37 ± 0.4 | 0.0001 |  |
| Creatinine ± SD (mg/dL) | 0.83 ± 0.4 | 0.82 ± 0.2 | 0.834 |  |
| Platelet count ± SD (x 10^9^/L) | 164 ± 72 | 239 ± 70 | 0.0001 | OR 0.985 (95%CI 0.981-0.988); p=0.0001 |
| Ferritin ± SD | 172 ± 219 | 207 ± 229 | 0.113 |  |
| [ALT/ULN / ALP/ULN] ratio | 3.49 ± 4.72 | 3.96 ± 4.16 | 0.001 | OR 0.89 (95%CI 0.82-0.98); p=0.016 |
