# Supplementary material for: The biochemical pattern defines MASLD phenotypes linked to distinct histology and prognosis
Source: J Gastroenterol. 2024 Apr 15;59(7):586–97. doi: 10.1007/s00535-024-02098-8 (PMC11217049; doi:10.1007/s00535-024-02098-8)
Supplement: Supplementary file 4 — Supplementary file4 (DOCX 13 KB) [file 535_2024_2098_MOESM4_ESM.docx]

| **Initial / Final** | Hepatocellular pattern | Mixed pattern | Cholestatic pattern |
| --- | --- | --- | --- |
| Hepatocellular pattern | 57.4% (128/223) | 35.4% (79/223) | 7.2% (16/223) |
| Mixed pattern | 22.8% (103/452) | 59.3% (268/452) | 17.9% (81/452) |
| Cholestatic pattern | 6.5% (15/230) | 37% (85/230) | 56.5% (130/230) |
